# Centre for the Promotion of Physical Activity and Health (CAPAS-City): A Pyrenean Cross-Cultural Structure to Lead the Way in the Design, Implementation, and Evaluation of Multilevel Physical Activity Interventions

**DOI:** 10.3390/ijerph16193631

**Published:** 2019-09-27

**Authors:** Javier Zaragoza Casterad, Javier Sevil-Serrano, Julien E. Bois, Eduardo Generelo, Léna Lhuisset, Alberto Aibar-Solana

**Affiliations:** 1 Department of Didactics of the Musical, Plastic and Corporal Expression, Faculty of Social Sciences and Humanities, University of Zaragoza, 22003 Huesca, Spain; zaragoza@unizar.es (J.Z.C.); generelo@unizar.es (E.G.); aibar@unizar.es (A.A.-S.); 2 Department of Didactics of the Musical, Plastic and Corporal Expression, Faculty of Health and Sport Sciences, University of Zaragoza, 22001 Huesca, Spain; 3 University of Pau & Countries of Adour, e2s UPPA, MEPS, Tarbes, France Quartier Bastillac, 65000 Tarbes, France; julien.bois@univ-pau.fr (J.E.B.); lena.lhuisset@univ-pau.fr (L.L.)

**Keywords:** physical activity, active commuting, leadership, disadvantaged populations, partnerships, stakeholders, multilevel, social-ecological model, health

## Abstract

This study describes a Pyrenean cross-cultural structure called Centre for the Promotion of Physical Activity and Health (CAPAS-City) that was created to promote physical activity (PA) in Huesca (Spain) and Tarbes (France). The main aim of this centre is to lead the way in the design, implementation, and evaluation of multilevel PA interventions to improve their efficacy and sustainability inside the city. CAPAS-City responds to the main challenges related to multilevel PA interventions, through six guiding principles: (1) promoting sustainability, (2) playing a leadership role, (3) promoting multisectoral partnerships, (4) using evidence-based strategies, (5) promoting integrated knowledge translation, and (6) using a participatory research approach. Five multilevel PA interventions were designed in both cities by CAPAS-City with these principles in mind. Through the example of the Annual MOT Test adapted to bikes, we also illustrate one practical application of the use of these principles, following the Social-Ecological approach, in which the main agents of influence are involved at different levels to encourage cycling. According to the promising results found in this study, CAPAS-City appears to be a structure that is able to respond to the main needs and challenges of multilevel interventions to increase PA levels in the whole population of both cities.

## 1. Introduction

It is widely established that regular physical activity (PA) provides physical, mental, and social health benefits for people of all ages [1,2]. However, 27% of adults and 80% of adolescents worldwide do not meet PA recommendations [3,4]. Results from Spain’s and France’s 2018 Report Cards on PA for children and youth, revealed that only 31% of Spanish males and 14.9% of Spanish females met PA recommendations [5], while in France only 23% reached them [6]. Promoting PA is, therefore, one of the key priority areas in public health promotion both for national entities, with the Prevention and Health Promotion Strategy (Spain), or the Nutrition and Health National Plan (France), and for international entities, with the PA strategy for the World Health Organization (WHO) European Region 2016–2025, and the WHO Global Action Plan on PA 2018–2030.

Although a lot of PA policies and PA intervention programmes have been developed to improve PA levels, effect sizes are usually small or even non-significant both in youths [7,8] and in adults [1]. Moreover, the effectiveness of PA intervention programmes in the long-term maintenance of PA also shows a small effect size in youths [9,10] and adults [11]. Understanding the correlates and determinants of PA behaviour becomes, therefore, essential to design and implement effective PA interventions. Given that PA is affected by multiple factors (i.e., intrapersonal, interpersonal, community, and policy) [1,12] across the life span, it may not be surprising that single level interventions are less effective in increasing PA levels than multilevel interventions [13]. The use of the Social-Ecological Model (i.e., is a comprehensive public health framework that recognizes all these multiple levels of influence on individual health behaviours), is, therefore, fully warranted to design multilevel PA interventions, which have been considered as one of the most promising strategies to enhance initiation and long-term maintenance of PA [14]. Despite growing evidence that suggests that multilevel PA interventions are more feasible, effective, and sustainable, there is a limited number of this type of interventions to date. It is therefore necessary to tackle some operational and empirical needs and challenges to design, implement, and evaluate multilevel PA interventions, to improve evidence-based practices in the real world [15].

### 1.1. Needs and Challenges Related to Multilevel Physical Activity Interventions

Although a deeper understanding of multilevel PA interventions has advanced over the last years, it is still necessary to overcome some needs and challenges. Sustainability is often defined as the maintenance of an intervention after the external researchers have left it [16]. PA interventions have traditionally focused exclusively on the intervention effects rather than on variables affecting their sustainability. The sustainability of multilevel PA interventions has been considered one of the most significant challenges to ensure long-term PA maintenance [17]. Lack of sustainability may be due to several reasons such as the fact that the intervention programme is implemented exclusively by external researchers. Moreover, multilevel interventions require a considerable amount of additional financial and temporary resources to attain multiple levels of socio-ecological systems [14,18]. The lack of theoretical behaviour change frameworks in the design of multilevel PA interventions [19], and the need to mobilize knowledge about evidence-based strategies from research to practice should be faced as one of the major challenges [20]. This type of intervention should also be adapted to each local context and the real world because there is no single or exclusive multilevel PA intervention solution [21]. Local needs and preferences as well as policy-makers’ agendas should be considered to ensure the effectiveness and sustainability of multilevel PA interventions [22]. Given that multilevel PA interventions are mostly conducted by research groups or by academic institutions [23], fostering a collaborative approach, which can drive connections between expertise from multiple disciplines and settings (i.e., teachers, researchers, local actors, clinicians, policy-makers, etc.), is required. Consequently, these connections will enhance the feeling of ownership and sustainability of the interventions [14,24]. Therefore, the ideal implementation of multilevel PA interventions requires leadership [25] to develop, discuss, coordinate, and sustain multidisciplinary partnerships [14]. Challenges in terms of sustainability, funding, theory, design, coordination, implementation, evaluation, and leadership of multilevel PA interventions should, therefore, be tackled [26].

### 1.2. Building Healthy, Liveable, and Sustainable Cities

In 2018, the WHO developed a new global action plan, within the new opportunities and agenda set by the 2030 Sustainable Development Goals, to increase the proportion of people who meet PA recommendations to 15% of the worldwide population. The Global Action Plan on PA 2018–2030 defined four strategic objectives: to create active societies, active environments, active people, and active systems. A policy plan, in which PA plays a significant role, should foster the design, implementation and evaluation of national PA plans for each country [27]. Moreover, as cities continue to grow in size, governments from the WHO European Region have recognized the need to provide supportive PA environments to ensure that all people can be physically active in the city context, particularly the least active populations [27]. According to the Spanish Ministry of Health, Social Services and Equality (2015), and the French National Institute of Statistics and Economical Studies (INSEE, 2016), at least 70% of the population in Spain live in towns with more than 20,000 inhabitants, while in France, 50% of the population live in towns with more than 10,000 inhabitants. Cities, and, particularly, neighbourhoods, workplaces, or schools, are key settings that might help to achieve the WHO’s four strategic objectives and, consequently, promote PA levels in the whole population [28]. For example, schools represent an excellent setting because they reach almost all children and adolescents and provides the opportunity to adopt a whole-of-school approach that involve educative agents such as families, teachers or peers. It also offers the possibility to develop strategies via curricular (e.g., physical education, school break, etc.) and non-curricular channels (e.g., family involvement, extracurricular PA activities). Therefore, designing healthy, liveable, and sustainable cities is, therefore, one of the main challenges of the WHO, in order to make people more active in a healthier world. To do this, multilevel community-based interventions, which address multiple levels of influence using a “system-based” holistic approach, may be required [27].

## 2. Centre for the Promotion of Physical Activity and Health (CAPAS-City)

CAPAS-City responds to the main needs and challenges of multilevel PA interventions in the cities of Huesca (Spain) and Tarbes (France), aiming to increase PA levels in the whole population of both cities.

### 2.1. Partners

The Centre for the Promotion of Physical Activity and Health (CAPAS-City; https://CAPAS-c.eu/) is a Pyrenean cross-cultural structure (Spain/France) which is composed by four partners, specifically the city councils of Huesca (Spain) and Tarbes (France), and two research groups with expertise in multilevel PA interventions from the University of Zaragoza (Spain), and the University of Pau and Pays de l’Adour (France), respectively. Education, sport, and governmental health services of both territories are also included in CAPAS-City as collaborators.

CAPAS-City has two different sites, one in each country, specifically in the cities of Huesca and Tarbes. Huesca and Tarbes are two mid-sized (around 50,000 inhabitants) twin cities situated close to each other (i.e., 220 km by road), but separated by the Pyrenean Mountains. Huesca and Tarbes have similar local needs and characteristics, such as population distribution, number of inhabitants, extension, local services, etc. Since 1964, the City Councils of these two twin cities have jointly developed community projects with the idea of opening up borders, and encouraging the development of cross-cultural relationships. Simultaneously, both universities have also held plenty of academic conventions, and conducted different common research projects about multilevel PA interventions. This traditional and regular cooperation facilitates the development of new cross-border-research-projects.

### 2.2. Funding

CAPAS-City was funded by the European programme for territorial cooperation and sustainable development of cross-border regions (Spain, France, and Andorra in this case) called POCTEFA 2014–2020. The four partners applied for common funding to create a Pyrenean cross-border centre for, initially, 3 years and a half, from July 2016 till December 2019.

### 2.3. Organizational Structure

**Figure 1:**
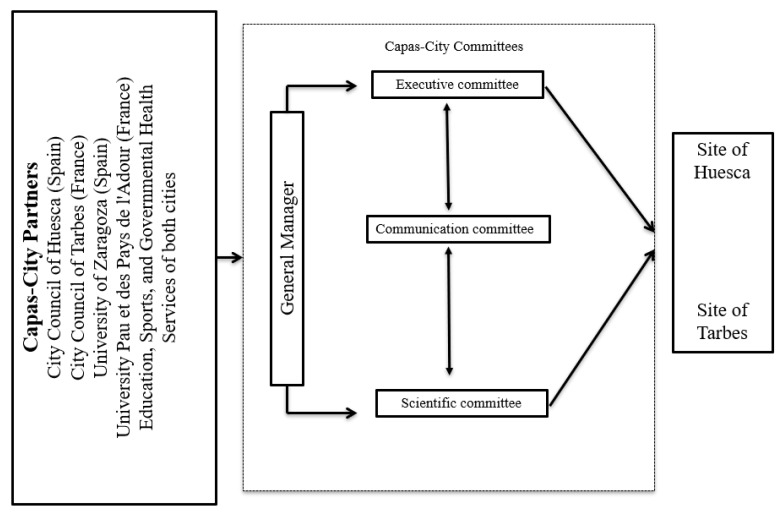
Organizational structure of CAPAS-City.

The organizational structure of CAPAS-City, is composed by members of the two sites. These members are distributed in different committees (not mutually exclusive) such as: the executive committee (whose role is to address issues that affect the organizational structure, applying for project funding to ensure the sustainability of CAPAS-City and to coordinate the other two committees of this organizational structure), the scientific committee (focussing on the design, implementation, coordination, and evaluation of multilevel PA interventions), and the communication committee (responsible for disseminating evidence-based practices, and translating knowledge into practice for the general population, through the website and social media, as well as developing PA resources and materials, organizing congresses, symposiums, and events). All committees meet regularly four times a year, and are coordinated by a general manager, in order to coherently respond to the interests and needs of both cities in terms of PA promotion. The organizational structure of CAPAS-City is shown in Figure 1.

### 2.4. Aim, Target Population, and Philosophy of CAPAS-City

Guided by the Social-Ecological framework, CAPAS-City has been created to overcome the main challenges related to multilevel and complex interventions [14,29]. The main aim of CAPAS-City is to lead the design, implementation, and evaluation of long-term multilevel PA interventions in both cities, to improve their efficacy and sustainability. Thus, this centre plays a leadership role among the multiple stakeholders involved in promoting PA in Huesca and Tarbes, to encourage the establishment of common synergies among them. It also reinforces and potentiates the long-term effect of multilevel PA interventions. Five multilevel PA interventions in schools and community settings (i.e., Paths of the Pyrenees, Promoting Active Transport to school (ProATs), Pio Keeps Moving, Move at School, and Move in your Suburb; for a further description, see Section 3.2 of this manuscript) were designed in both cities by CAPAS-City, according to the interests and needs of the population and of all the stakeholders. All these multilevel PA interventions organized by CAPAS-City consider the correlates and determinants of PA that co-exist and affect PA from multiple levels of influence across the life span [30]. Given that some community and policy levels of the Social-Ecological Model require time to build environmental changes and local policies related to PA, one of the premises of CAPAS-City is that all multilevel PA interventions should have a minimum duration of at least nine months.

Although CAPAS-City aims to influence the whole population of both cities, multilevel PA interventions promoted by this centre focus particularly on priority population groups (i.e., children, adolescents, disadvantaged population). Young people and people from disadvantaged population groups are more likely to not meet PA recommendations and to experience more detrimental health outcomes [4]. Moreover, early years interventions are recommended because young people who adopt a healthy lifestyle are likely to maintain it throughout life and achieve benefits for their adult health [31]. Therefore, improving participation in PA among these priority population groups becomes a public health priority to reduce health inequalities [32].

As far as the philosophy aspects of CAPAS-City are concerned, it must be noted that research literature suggests that the way in which PA is promoted may influence the initiation and long-term maintenance of PA [33]. It has to be highlighted that the main focus of the CAPAS-City PA promotion model is not on changing physical appearance, preventing or treating diseases, or sport performance and competition. Given that other variables such as autonomous motivation and enjoyment have been considered a vehicle for long-term PA participation in all age ranges, the philosophical conception of CAPAS-City aims to design meaningful and enjoyable activities in order to provide positive PA experiences to the whole population [33]. In addition, although multilevel interventions designed by CAPAS-City mainly focus on PA, other health-related behaviours are also promoted to maximize health benefits [34].

Embedded in the main aim and the philosophical conception of CAPAS-City mentioned above, this centre has developed six guiding principles to cope with the current main challenges related to multilevel and complex interventions [14,29]. The following guiding principles were used to design, implement, and evaluate multilevel PA interventions.

### 2.5. Guiding Principles for Multilevel PA Interventions

#### 2.5.1. Promoting Sustainability

Several systematic reviews that have analyzed the long-term effectiveness of PA interventions in both youth and adults, revealed that intervention effects diminish over time [9,10,11]. This decrease could be due to the lack of sustainability of PA intervention programmes. Sustainability requires the empowerment of the main agents involved in the implementation of school-based and community interventions (e.g., policy-makers, school management teams, school teachers, families, public health practitioners, staff in local community, etc.) to sustain the intervention effects over time [35]. That is the reason why CAPAS-City provides continuous training for school community agents and all the local community members in the design and development of multilevel PA interventions. In addition, CAPAS-City provides training in motivational techniques to promote need-supportive environments (i.e., autonomy, competence, and relatedness support strategies) for stakeholders, partnerships, and members of the school community, on how PA should be promoted to achieve positive PA experiences that could influence long-term PA behaviour. All this information is provided by CAPAS-City through a variety of digital and printed resources and materials, available on the website.

#### 2.5.2. Promoting Integrated and Multisectoral Partnerships

An integrated, multidisciplinary, and intersectoral approach is required to increase PA at population level [27]. Multilevel interventions must incorporate the participation and expertise of different agents from a wide range of disciplines [14,29]. CAPAS-City promotes the participation of multisectoral partnerships involved in the promotion of PA at different levels, such as education, health practitioners from multiple community settings, policy, research, etc., to effectively address the most challenging PA issues. Regular meetings and fluent information-sharing sessions are held with stakeholders and partnerships, who are strongly encouraged to establish warm relationships, priorities, needs, and common preferences.

#### 2.5.3. Playing a Leadership Role

Given the complex and long-term nature of multilevel PA interventions, the existence of a leadership group [25] to recruit, coordinate and sustain multidisciplinary partnerships becomes of paramount importance [14,29]. Several studies have revealed that multisectoral and partnership-based approaches are required to achieve a greater sustainable impact of interventions [36]. Consequently, CAPAS-City, which is coordinated by a general manager through three interconnected organizational structures, is basically aimed at promoting the establishment of synergies among key stakeholders from multiple sectors, involved in the promotion of PA. 

#### 2.5.4. Using Evidence-Based Strategies

All multilevel PA interventions led by CAPAS-City are guided by theoretical frameworks, specifically by the integration of the Social-Ecological Model and the Self-Determination Theory (SDT) as has been suggested by previous studies [37]. The Social-Ecological Model is a comprehensive public health approach that recognizes multiple levels of influence of PA behaviour [30], while the SDT is a macro-theory of motivation that assumes that the satisfaction of autonomy, competence, and relatedness affects people’s motivation and, consequently, PA behaviour [38]. Previous systematic reviews and meta-analysis showed that SDT is the social-cognitive theory that explained the greatest amount of variance in PA (i.e., 37%) [39] and has been shown effective in increasing PA levels in a wide range of populations [40]. CAPAS-City is based on evidence-based knowledge and research expertise [40], using strategies that have already been effective in improving PA and other health-related behaviours in multiple settings (e.g., schools, community, etc.) [40,41,42,43]. Nevertheless, it should be highlighted that CAPAS-City conducts an initial evaluation of each context and sample, to adapt all these evidence-based strategies to their local and individual needs.

#### 2.5.5. Promoting Integrated Knowledge Translation

To translate scientific knowledge into practice, researchers must disseminate the results of their studies to the whole society in an understandable and meaningful way [44]. Some of the strategies proposed by Giles-Corti et al. [45] are used by CAPAS-City to facilitate this translation of knowledge: positive relationships between researchers, policy-makers, and practitioners, collaborative interdisciplinary research projects, and understanding the needs and preferences of the policy-makers and practitioners. Following the tips provided by Tripathy et al. [46] or the WHO [47], to improve the visibility of research findings, CAPAS-City disseminates the results and conclusions of research through a range of social media channels (e.g., local television, radio, local newspaper, twitter, Facebook), national and international events (e.g., symposiums, congresses, workshops), short overviews (e.g., policy briefs, infographics, technical reports), or open access journals or repositories (e.g., ResearchGate, Academia, Publons, Orcid).

#### 2.5.6. Using a Participatory Research Approach

CAPAS-City gives voice to the community, policy-makers, and research participants, using a participatory action research approach to address problems and needs that emerge from the community [29,48]. All multilevel PA intervention programmes designed by CAPAS-City incorporate regular self-evaluation sessions to adapt the intervention programme itself. Mixed method designs are particularly useful in this type of participatory action research approaches [49]. CAPAS-City combines qualitative and quantitative research methods to provide a further understanding of the local needs and priorities of the community and, consequently, adapt the intervention to the local context. Through qualitative techniques, CAPAS-City empowers policy-makers, and all members of the community and school, in the design, implementation, and evaluation of multilevel PA intervention programmes, to foster in them a sense of ownership and responsibility and, consequently, to generate changes in policy and/or practice.

## 3. CAPAS-City: Organizational Design and Multilevel PA Interventions

### 3.1. General Organizational Design

**Figure 2:**
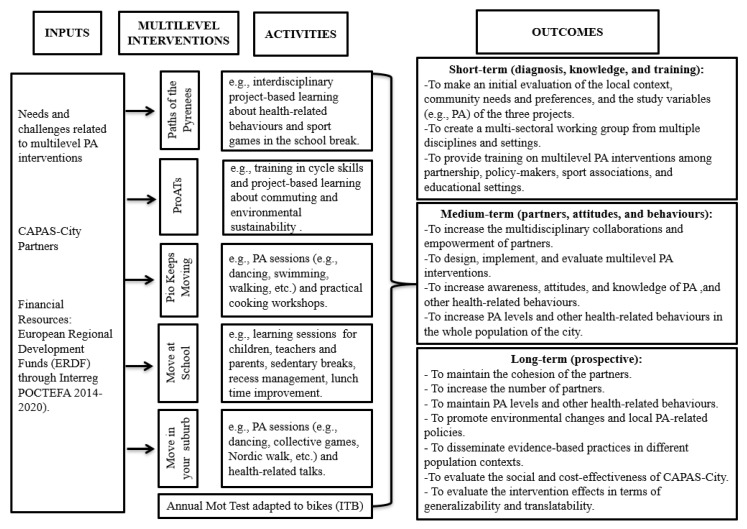
Summary of the organizational model used by CAPAS-City: Inputs, multilevel interventions, activities, and outcomes.

CAPAS-City is guided by a Social-Ecological framework to lead the way in the design, implementation, and evaluation of multilevel PA intervention programmes. To do this, different inputs can be used to obtain several expected outcomes. CAPAS-City is responsible for organizing those inputs, through different multilevel PA interventions and their subsequent activities, to achieve the expected outcomes in the short-, medium- and long-term. In Figure 2, we present the organizational model used by CAPAS-City to promote PA.

As shown in Figure 2, the inputs of CAPAS-City have been used to design the five multilevel interventions (i.e., Paths of the Pyrenees, ProATs, Pio Keeps Moving, Move at School, and Move in your Suburb) to promote PA levels and other health-related behaviours. These five interventions are comprised of three common phases: a diagnostic assessment phase (to identify the prevalence and compliance with PA, and other health-related behaviour recommendations), an intervention phase (to promote PA and other health-related behaviours), and a final assessment phase (to evaluate the short-, medium-, and long-term effects of multilevel interventions). Although these multilevel interventions develop specific activities, there are also common strategies, such as the Annual MOT Test adapted to bikes (ITB, the Spanish acronym for Inspección Técnica de Bicicletas) (see further details in Section 4), which addresses the entire population of both cities. All these interventions are proposed to generate a series of short-, medium- and long-term outcomes related to the promotion of PA. To better understand this organizational model, the five main multilevel PA intervention programmes deserve to be further explained in the following point.

### 3.2. Multilevel PA Interventions

The main characteristics of multilevel PA interventions conducted by CAPAS-City are summarized in Table 1. It has to be noted that all study variables were measured before and after the intervention programmes.

#### 3.2.1. Paths of the Pyrenees Intervention

”Paths of the Pyrenees” is a multicomponent school-based intervention programme to promote multiple health behaviours such PA, sedentary time, sleep duration, (un)healthy diet, and alcohol and tobacco substance consumption among a convenience sample of adolescents aged 12 to 14 years old. This intervention design adopts a system-based approach that fosters the empowerment not only of all members of the school community (i.e., students, families, and school management teams), but also of public health practitioners and policy-makers, to create a more sustainable and healthier school environment. This intervention is mainly conducted by school teachers through curricular actions (i.e., tutorial action plan, interdisciplinary project-based learning, and school break), and extracurricular actions (i.e., family involvement, institutional and non-curricular activities, and dissemination of health information and events) (for a further review see [50]). In this intervention, CAPAS-City is responsible for providing teacher training on multilevel PA interventions, benefits, risks, guidelines, and prevalence of PA, and other health-related behaviours, and need-supportive teaching strategies. This centre is also responsible for coordinating all curricular and extracurricular actions within the school community staff and the local context, and evaluating their effects. All actions and strategies of the “Paths of the Pyrenees” intervention programme are also being co-developed and co-supervised by CAPAS-City.

#### 3.2.2. ProATs Intervention

“ProATs” is a multicomponent school-based intervention programme to promote active transport to school (ATS) in a convenience sample of children aged 10–12 years old. This intervention programme is conducted by school teachers through a project-based learning project from an interdisciplinary perspective across different areas. The WHO Health Promoting Schools framework is used. This intervention is characterized by, (i) the connection between the school and the local community (i.e., participation of different school members and community agents), and (ii) the leadership of the physical education teacher with the help of a facilitator (i.e., a member of Capas-City who has been appointed to coordinate the programme). ProATs is comprised of three intervention components (i.e., formal curriculum, non-formal curriculum, and community) that integrate the most promising PA strategies identified in literature [43]. To design and implement this programme, teachers participated in a training workshop conducted by CAPAS-City at the beginning of the intervention. CAPAS-City is also responsible for coordinating, supervising, and evaluating all actions developed in the school and local community.

#### 3.2.3. Pio Keeps Moving Intervention

“Pio Keeps Moving” is a community-based participatory intervention designed to promote PA, healthy eating, and other health-related behaviours and outcomes among a convenience sample of disadvantaged adult women, particularly among Roma women. Disadvantaged population groups are defined as those described as low socioeconomic status, low income or low education. This multilevel intervention is conducted to address one serious health problem that was detected by the medical service in a disadvantaged neighbourhood (i.e., is a deprived area with low socioeconomic status and unemployment). A high proportion of the adult population that live in this disadvantaged neighbourhood are overweight or obese, and their children also report alarming obesity rates. 

Different community agents such as doctors, nurses, psychiatrists, teachers, social workers, nutritionists, and PA professionals contribute to jointly designing and implementing this intervention programme. This intervention is based on a participatory action research approach that permits constantly modifying the programme according to the needs and preferences of the target population. One of the main aims of this programme is to help adult Roma women to develop autonomy and a pro-active behaviour that will facilitate self-management and the long-term maintenance of a healthy lifestyle. All actions of the “Pio Keeps Moving” programme are being coordinated and supervised by CAPAS-City.

#### 3.2.4. Move at School Intervention

“Move at School” is a multilevel school-based intervention programme aimed at increasing PA levels, physical fitness, and academic achievement, and also at decreasing sedentary time among a convenience sample of children, aged from 6 to 11 years old, located in disadvantaged neighbourhoods. This intervention aims to involve all the school community agents (i.e., children, teachers, parents, and after-school management teams) as well as the city council services. This intervention is conducted by a doctoral student from CAPAS-City who provided knowledge and awareness of PA and sedentary time to children, teachers and parents (e.g., benefits, risks, prevalence, guidelines, etc.), teacher training (e.g., active classroom, sedentary breaks, etc.), and support for PA (e.g., games). All these actions are co-developed with the different school community agents (i.e., children, teachers, after-school agents, and city council services) to ensure their sustainability.

#### 3.2.5. Move in Your Suburb Intervention

“Move in your Suburb” is a community-based intervention designed to increase PA levels, physical fitness, and well-being, and to decrease sedentary time and loneliness in disadvantaged neighbourhoods. A convenience sample of disadvantaged adult men and women participated in this study. A high proportion of the population living in these neighbourhoods is inactive and sedentary, and have a high prevalence of non-communicable diseases. This programme involves social workers, health practitioners, physical educators, and all sports associations of this neighbourhood in order to design, implement, and sustain the different strategies carried out. CAPAS-City has designed different PA sessions adapted to the particular characteristics of this population. The intervention is divided into three phases. In the first phase, the PA sessions are supervised by a physical educator from CAPAS-City. In the second phase, people participated in free-time and organized PA sessions taught either by the physical trainer or by different sports associations of this neighbourhood. Finally, in the third phase, people were accompanied by the physical trainer to the sports association of their choice to maintain their PA goals. CAPAS-City coordinates and supervises this multi-component programme and evaluates the effects of the three phases.

## 4. “ITB”: An Example of a Strategy Embedded in a Socio-Ecological Framework and Conducted by CAPAS-City to Promote Cycling in the City

Promoting walking, cycling, and other modes of active commuting is one of the objectives developed by the WHO in the Global Action Plan on PA 2018–2030 to create active societies and active environments [27]. It is well-known that active commuting (i.e., walking and cycling) is affected by multiple factors at multiple levels of influence [51]. The Social-Ecological framework points out that interventions that address multiple levels of influence should be more effective and sustainable in promoting active commuting than intervention programmes based solely on one level [52]. The current challenge in literature is to identify evidence-based strategies from different levels with the aim of increasing cycling as an active mode of transport in the entire population [53,54].

In this section we would like to introduce an example of a strategy whose design is based on socio-ecological principles and led by CAPAS-City, called the ITB, which has been developed from 2016 to date. This activity tries to encourage cycling in the city of Huesca by way of a multilevel intervention.

**Figure 3:**
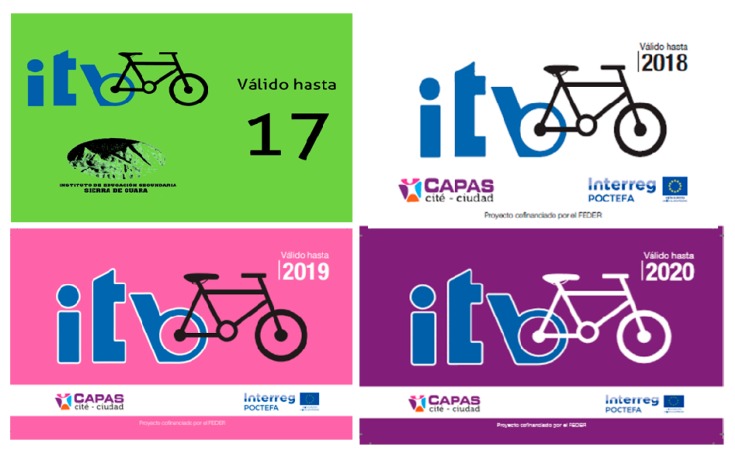
ITB certificates for 2016, 2017, 2018, and 2019.

In general terms, the ITB is an annual test of bicycle safety, roadworthiness aspects, and cycling-related skills and knowledge. This activity addresses the whole population of the city and young people (primary, high school, and university students), in particular, as they are considered a priority population behaviour change [7]. Participating in the ITB is completely free. This activity mainly integrates a series of workshops in which mechanical bicycle problems are identified, and basic roadworthiness and cycling-related skills are evaluated. Participants who meet all basic criteria from the different workshops obtain the ‘ITB certificate’ (see Figure 3 for the 2016, 2017, 2018, and 2019 certificates), which is valid for one year. This certificate is not a mandatory requirement for circulating in the city. It just symbolizes that the bicycle is ready for use and that the participants have the main cycling-related skills and knowledge necessary to move around the city with safety and security. If the certificate cannot be issued, participants are offered the possibility of repairing their bicycles with an economic discount at any bicycle shop in the city that collaborates annually with this activity. It should also be noted that, due to the inclusive nature of the ITB (i.e., addressing the whole city population), wheelchairs, tandems, and other adapted vehicles can also be repaired and obtain the certificate. Unlike the usual standardized Ministry of Transport Test (MOT) to certify vehicles, the ITB does not aim to be an isolated and decontextualized activity aimed at just inspecting or repairing bicycles. This activity is an annual reference action in the city, empowering citizens to use bikes to commute in the city in a sustainable, autonomous, and efficient way. All workshops are taught by multidisciplinary teams comprised mainly of the scientific committee from CAPAS-City, local police officers, the Directorate General for Traffic, and graduates in Physical Activity and Sports Sciences.

**Figure 4:**
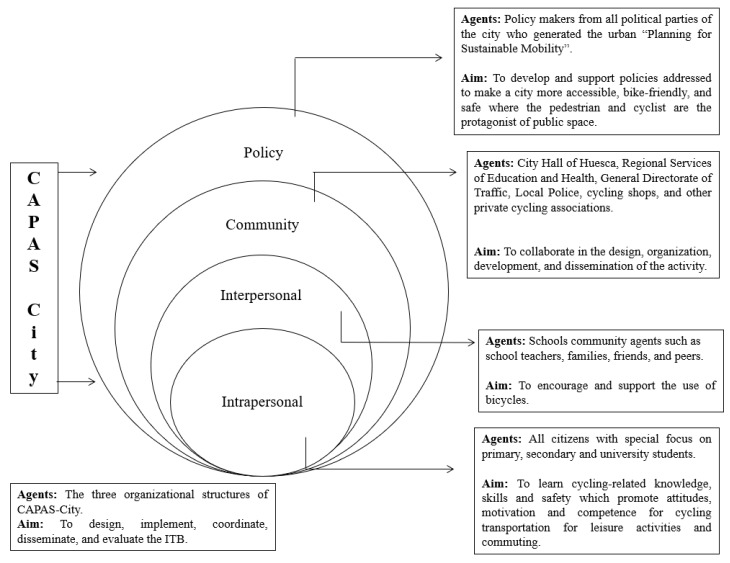
Agents involved and objectives at each level of influence of the Social-Ecological approach for the ITB.

The ITB is a clear example of a multilevel PA intervention, using a multisectoral and partnership-based approach upheld by CAPAS-City, which integrates the main agents involved in the promotion of active transport, particularly cycling. Using a Social-Ecological approach, the main agents of influence at different levels (i.e., individual, interpersonal, community, and political) as well as their specific objectives in the ITB, were identified (see Figure 4). Below, we describe the main features of this global strategy for each level of influence included in the Social-Ecological framework. Nevertheless, it should be highlighted that, from our point of view, the ITB also enhances stakeholders’ awareness of their potential influence for the promotion of cycling in the city.

At an individual level, all citizens, and especially young people, are invited to participate in this activity. To involve the school context, all students at the city schools are annually invited to obtain the ‘ITB certificate’ as a prior and recommendable step to start their cycling teaching unit. Thus, teachers can also verify that students’ bicycles are in perfect conditions to begin that teaching unit. Both actions, the ITB, plus the cycling unit, should improve the skills, knowledge and, consequently, the autonomy and confidence in relation to the maintenance and safe use of the bike as a means of transport [55]. The ITB is also expected to facilitate the acquisition of attitudes, motivation, and competence for cycling as a transport means for the whole population.

At an interpersonal level, and given that students are one of the main target populations, the ITB involves different agents from the educational community, who may enhance and support cycling as a mode of transport. School teachers, school management teams, families, and students from different educational centres are actively involved in the development of the ITB. A school community training programme about strategies to promote cycling in the city is required of the school members and local community (i.e., teachers, university students, etc.) to collaborate in the ITB. Moreover, an initial training of PE teachers who teach the cycling unit is also required to develop teachers’ cycling-related knowledge. Prior to completion of the ITB, different meetings with school management teams, press conferences, advertising in local media, and advertising posters around the city, are conducted to encourage the school community, not only to participate in the ITB, but also to promote and support the use of the bicycle as a sustainable and active mode of transport. At this point, we would like to highlight the key role played by a group of students from a vocational training school specialized in motor vehicle maintenance and repair. Guided by the adoption of a service-learning project, these students oversee the examination and repair of bicycles during the ITB. Although these students have a high initial knowledge about mechanics, they also carry out specific training in cycling mechanisms and cycling-related knowledge, to acquire the necessary autonomy and competence to be responsible for managing the different ITB workshops. 

At community level, different local agents from the city such as the City Council of Huesca, the Regional Education and Health Services, the Directorate General for Traffic, the Local Police, cycling shops, and other private cycling associations aim to actively collaborate in the design, organization, development, and dissemination of the ITB through several annual meetings. It should be noted that the cycling shops provide economic discounts for mechanical assistance to any bike whose repair has been suggested after the ITB in order to obtain the ‘ITB certificate’.

At policy level, the local public administration (e.g., different political parties from the City Council of Huesca), different private cycling-related companies (e.g., bicycle shops), and other social agents of the city (e.g., cycling clubs) signed the urban ‘Planning for Sustainable Mobility’ in 2015. This common agreement aims to develop policies in order to favour a more accessible, friendly, and safe city, where pedestrians and cyclists are expected to be the main users of the city’s public spaces instead of drivers of motorized vehicles. All these actions are being developed around two main principles: (a) changing the physical environment (e.g., improving the urban and interurban bike lane network, the distribution and quality of the public transport service, new bicycle parking areas and zoning regulations, etc.) to influence the population to primarily choose active modes of transport, and (b) changing people’s perception through active mobility initiatives, such as marketing campaigns and messages to promote the use of bicycles (e.g., guideline containing tips to use the bike and cycling skills training programmes). The ITB is perfectly placed in the second principle within the mobility plan that derives from the ‘Planning for Sustainable Mobility’. The ITB is a strategy that not only has local political support but is also expected to become part of the culture of the city.

The leadership role of CAPAS-City in the design, implementation, and coordination of the ITB becomes of paramount importance. CAPAS-City mainly promotes the establishment of synergies among multiple stakeholders at different levels (i.e., individual, interpersonal, community, and political) that are directly or indirectly involved in the promotion of cycling in the city. Moreover, CAPAS-City also aims to evaluate ITB participation and other cycling-related skills, barriers, and knowledge. For instance, the evaluation of the number of annual participants at the activity, or the identification of the main barriers to commuting by bike in the city, are some of the actions conducted by CAPAS-City.

## 5. Preliminary Results

The long-term effects of the five multilevel PA interventions designed by CAPAS-City will be available at the end of 2019. We still have to wait to know the role of CAPAS-City in achieving greater and more efficient multilevel PA interventions in both cities. Nevertheless, some preliminary findings of the five multilevel PA interventions (i.e., Paths of the Pyrenees, ProATs, Pio Keeps Moving, Move at School, and Move in your Suburb) are highlighted. It should be noted that some results from “Paths of the Pyrenees” programme have already been published [39], but results from “ProATs”, “Pio Keeps Moving”, “Move at School”, and “Move in your Suburb” programmes are still under consideration for publication. The two-year follow-up post-intervention results of “Paths of the Pyrenees” are also under construction. Below, we present the more relevant preliminary results in the five multilevel PA interventions designed by CAPAS-City.

*Paths of the Pyrenees*—Experimental school students showed a significant improvement in meeting PA, screen time, and sleep duration recommendations, as well as sedentary levels, (un)healthy food, and alcohol and tobacco consumption rates compared with control school students and their baseline values. It should be highlighted that a high effect size (η_p_^2^ = 0.40) was found in PA levels. Experimental school students reported a significant improvement in meeting PA guidelines compared to their baseline values (i.e., from 23% to 64.6%), and to control school students (i.e., from 18.8% to 64.6%). Experimental school students also reported significant improvements in almost all psychological correlates of PA that were measured (e.g., autonomy, competence, autonomous motivation, intention to be physically active, etc.), when compared to both control school students and their own baseline values.

*ProATs*—The experimental school group showed a significant increase in ATS (i.e., weekly light, moderate, and vigorous PA), as well as psychological variables associated with ATS (e.g., attitude, intention, and autonomy), when compared to both control school students (overall effect size (η_p_^2^) of 0.53) and their own baseline values.

*Pio Keeps Moving*—Disadvantaged adult women reported improvements in subjective and objective PA levels (weekly vigorous PA: from 0.40 ± 0.46 to 4.17 ± 3.14 min; d = −2.67; *p* = 0.008; R = −0.63), (un)healthy eating behaviours, and other health-related behaviours and outcomes compared to their baseline values. Particularly, through one-to-one semi-structured interviews and discussion groups, these women revealed the incorporation of new physical activities into their current lives, improvements in motor skills and physical fitness, intention to participate in PA, physical health benefits, body image changes, and the adoption of a healthy eating pattern. The health-related behaviours of the children and husbands of these women also seem to have benefited from the intervention thanks to the synergistic effect of different intervention strategies.

*Move at School*—Children from the experimental school reported a significant improvement in PA recommendations, going from 63% at baseline to 71% after the intervention programme while the control school only reached 66%.

*Move in your Suburb*—Participants from disadvantaged neighbourhoods (43 women and 15 men aged 48 ± 13 years old) reported significant improvements in physical fitness. After three months of PA sessions (phase 1), their leg strength significantly increased (*p* = 0.03). A further three months’ participation in PA sessions (phase 2) led to a significant improvement in leg strength (*p* < 0.001) as well as an increase in flexibility (*p* = 0.006), and endurance (*p* = 0.03). Participants also reported a significant improvement in their self-esteem, especially in their physical self-esteem (*p* = 0.04 after phase 1 and *p* = 0.02 after phase 2).

## 6. Discussion

Given the high percentages of inactive population in Spain and in France, the promotion of PA is one of the health priorities in these countries, particularly among priority populations such as students [5,6], and disadvantaged populations [4]. In this study, we describe a cross-cultural structure called CAPAS-City that is leading the design, implementation, and evaluation of multilevel PA interventions to improve their efficacy and sustainability in Huesca and Tarbes. The overall aim of CAPAS-City is to increase PA levels of the population in both cities.

Although no follow-up post-intervention has yet been developed, the preliminary results obtained in the five multilevel PA interventions (i.e., Paths of the Pyrenees, ProATs, Pio Keeps Moving, Move at School, and Move in your Suburb), designed by CAPAS-City, showed that all experimental groups reported significant improvements in PA levels, and other health-related behaviours and outcomes. Moreover, the positive impact of the interventions on motivational outcomes for leisure-time PA may promote the long-term maintenance of PA over time [56]. These results are particularly promising, because the interventions focussed on the least active populations [27] and were developed in a real-world context, by the main agents involved in the interventions in order to ensure their sustainability. Although it is difficult to specifically identify the principal cause of the effectiveness of these multilevel PA interventions, we strongly believe in the importance of the six aforementioned principles as key points for delivering successful projects in terms of PA promotion. The six guiding principles that support CAPAS-City could overcome some of the identified barriers related to multilevel PA interventions and, consequently, may enhance initiation and long-term maintenance of PA. It should be noted that these six principles of CAPAS-City, respond to the main needs and challenges related to complex multilevel PA interventions that have previously been identified in research literature [14,29].

As a remarkable result, it should be highlighted that a large effect size was observed in PA levels in the Paths of the Pyrenees, ProATs, and Pio Keeps Moving interventions. The effect sizes found are much better than other PA interventions conducted in adolescents [7,8] and socioeconomically disadvantaged groups [32]. Although it is difficult to specifically determine which specific intervention component or level of the Social-Ecological model was more effective, several explanations could be provided to account for these results. For example, the integration of two theoretical frameworks and evidence-based strategies, which have shown to be effective in improving PA levels, could be particularly useful to increase that behaviour [43,57]. These results contribute to a growing body of literature that suggests that developing multilevel interventions, incorporating changes or actions at different levels of the Social-Ecological model, could have greater effects on increasing PA levels than single level interventions [14].

The leadership role played by CAPAS-City to design, implement, and evaluate the multilevel PA interventions could facilitate the coordination of the different stakeholders that were involved in those interventions. On the one hand, CAPAS-City helped to create a multisectoral partnership in a city with multiple disciplines, expertise, and settings, to foster collaboration across and among all stakeholders at all levels. This intersectoral action for health could also contribute to enhancing stakeholders’ awareness about their important role in the promotion of PA and, therefore, reinforce the effectiveness and sustainability of these multilevel PA interventions. In line with our results, a previous 5-year community PA intervention conducted in Liverpool, and led by a broad range of agencies from a variety of disciplines, also showed an increase in PA levels in the whole population and, particularly, in disadvantaged neighbourhoods [55]. On the other hand, considering the needs and preferences of all the community (i.e., policy-makers, citizens, local actors, schools, stakeholders, and public health practitioners, etc.), CAPAS-City reinforced the sense of partnership in the PA agenda, empowering all the community, and adapting the intervention to the city context [22]. It is important to consider that although the leadership role of CAPAS-City could be similar in another local context, the multilevel PA interventions designed in the cities of Huesca or Tarbes have been addressed for those that are less likely to meet PA guidelines. Therefore, the six principles followed by CAPAS-City could have facilitated achieving the aim of this structure.

Long-term and community interventions, such as these five programmes designed by CAPAS-City, could contribute to achieving the target set for PA by the WHO Global Action Plan, and reduce health disparities. Although multilevel PA interventions should be adapted to each local context, the leadership role of CAPAS-City to design, implement, and evaluate these types of interventions may serve as a reference for other cities to create healthy, liveable, and sustainable cities. Initiatives that CAPAS-City fosters, such as the ITB activity, also seem to illustrate promising strategies at multiple levels of influence to promote cycling in the city, one of the most healthy and sustainable PA behaviours. Given that the most common barrier to PA is lack of time, both in youth and adults [57], cycling in the city has been considered an excellent opportunity to save time, while simultaneously incorporating PA into people’s everyday lives [58]. The long-term post-intervention effects of these multiple PA interventions will allow to evaluate their sustainability and cost-effectiveness and, consequently, the impact of CAPAS-City.

### Limitations and Difficulties

Several limitations should be considered. Firstly, participants from all the multilevel PA interventions were recruited through convenience sampling, which could introduce bias into the results. Secondly, considering the complexity and the multicomponent approach of multilevel PA interventions, we could not determine with precision which components, intervention level, principles of CAPAS-City, strategies or community social agents were more effective in increasing PA levels and other health-related behaviours. Thirdly, the preliminary results still do not allow to confirm the long-term maintenance of PA levels and other health-related behaviours, or the scope and sustainability of the three PA multilevel interventions. Finally, randomized controlled trials should be warranted to confirm our preliminary results. The main difficulties that CAPAS-City must cope with to lead the design, implementation, and evaluation of the multilevel PA interventions should be acknowledged. Firstly, these types of intervention programmes are time-demanding processes that require the contribution of numerous city departments, public-private partnerships, policy-makers, sports associations, and educational settings. Collaboration among all these partners takes time and requires considerable effort to reach common agreements. Nevertheless, we strongly believe that this difficulty also becomes a strength for all intervention programmes. Secondly, the involvement and empowerment of several stakeholders with different views about health and PA promotion have made leadership a difficult task. It is true that reaching consensus becomes more difficult when there are more stakeholders and more perspectives. However, we also believe that finding solutions that address everyone’s concerns and needs is a much better solution in the context of PA promotion. Finally, another difficulty was that policy-makers did not often involve CAPAS-City or other health professionals in participatory processes to build environmental changes and local policies related to PA. In addition, preferences and needs of policy-makers were not always in line with the main interests of the rest of the population and the evidence-based policies to promote PA. In line with the difficulties that were found in other studies [59], different ideologies and prejudices, policies that ignore scientific evidence, sectoral cultures, or short political timelines were also added difficulties for maintaining the local PA-related policies. Regular dialogue and consensus-building processes among policy-makers and different community agents become main points to address this type of difficulties.

## 7. Conclusions

The Social-Ecological framework seems to be a useful conceptual framework for designing and implementing complex and long-term multilevel PA interventions. The promising results of Paths of the Pyrenees, ProATs, Pio Keeps Moving, Move at School, and Move in your Suburb, led by CAPAS-City, seem to be perfect examples of effective multilevel PA interventions. Both these interventions and the ITB illustrate promising strategies at multiple levels of influence to increase PA levels in the whole population of both cities. Therefore, within this ecological framework, CAPAS-City becomes a cross-cultural structure able to contribute to the development of healthy, liveable, and sustainable cities and, specifically, to achieve the target set for PA promotion by the WHO Global Action Plan.

## Figures and Tables

**Table 1:** Characteristics of multilevel PA interventions conducted by CAPAS-City: aim, design/methods, sample ages, measures and instruments.

Project	Aim	Design/Method	Sample Ages	Measures and Instruments
Paths of the Pyrenees	To examine the effects of a school-based intervention on multiple health behaviours and other motivational outcomes	Quasi-experimental design (experimental and control group) One academic year/600 h	Adolescents aged 12–14 years	PA and sedentary time were measured by accelerometers. The other health-related behaviours were measured by using self-reported scales. Motivational outcomes from SDT framework were measured by using self-reported scales. Discussion groups were also conducted.
ProATs	To examine the effects of a school-based intervention on active transport to school and other motivational outcomes	Quasi-experimental design (experimental and control group) Two academic years/100 h (24 weeks)	Children aged 10–12 years	PA and sedentary time were measured by accelerometers Active transport was measured by accelerometers. Perceived barriers and motivational outcomes from SDT framework were measured by using self-reported scales. Focus groups among children, parents, and teachers were also conducted.
Pio Keeps Moving	To examine the effects of a community-based healthy lifestyle programme on PA, healthy eating, and other motivational outcomes	Community-based participatory action research 2 years/154 h 127 PA and nutrition sessions	Disadvantaged adult women (particularly adult Roma women), from 27 to 58 years old.	PA and sedentary time were measured by accelerometers. One-to-one semi-structured interviews and discussion groups were conducted to measure PA, healthy eating, other health-related behaviours and motivational outcomes.
Move at School	To examine the effects of a school-based intervention on PA, sedentary time, physical fitness, motivational outcomes and academic achievement	Quasi-experimental design (experimental and control group) One academic year	Children aged 6–11 years	PA and sedentary time were measured by accelerometers. Physical fitness was estimated based on the Eurofit battery test. Motivational outcomes from SDT framework were measured by using self-reported scales Academic achievement was assessed using standardized tests.
Move in your Suburb	To examine the effects of a community-based intervention on PA, sedentary time, physical fitness, well-being, loneliness, and motivational outcomes	One arm interventional study (experimental group only) 9 months (3 3–month phases)	Disadvantaged adult men and women (48.5 ± 13.5 years old)	PA and sedentary time were measured by accelerometers. Physical fitness was assessed with field tests. Physical self-perceptions, well-being, perceptions of loneliness, and motivational outcomes from SDT framework were assessed with self-reported scales.
